# Resource utilization and inpatient hospitalization costs associated with thromboembolic events among patients with polycythemia vera

**DOI:** 10.1093/oncolo/oyaf001

**Published:** 2025-03-04

**Authors:** Jingbo Yu, Julie Gayle, Ning Rosenthal, Harold Brown, Evan Braunstein, Naveen Pemmaraju

**Affiliations:** Incyte Corporation, Health Economics and Outcomes, Wilmington, DE 19803, United States; PINC AI^™^ Applied Sciences, Premier Inc., Charlotte, NC 28277, United States; PINC AI^™^ Applied Sciences, Premier Inc., Charlotte, NC 28277, United States; PINC AI^™^ Applied Sciences, Premier Inc., Charlotte, NC 28277, United States; Incyte Corporation, Health Economics and Outcomes, Wilmington, DE 19803, United States; Department of Leukemia, The University of Texas MD Anderson Cancer Center, Houston, TX 77030, United States

**Keywords:** thrombosis, embolism, polycythemia vera, inpatient, hospital, cost

## Abstract

**Background:**

Healthcare resource utilization (HCRU) and costs are often elevated in patients with polycythemia vera (PV), and this patient population has an increased risk of developing thromboembolic events (TEs). This study describes HCRU, costs, and mortality during TE-related hospitalizations among patients with PV in a contemporary real-world setting in the United States.

**Patients and Methods:**

This retrospective cohort study included adult inpatients with PV and TE discharged from 623 hospitals between January 1, 2017, and June 30, 2020 with a 2-year follow-up period after the first TE-related (index) hospitalization. Data were abstracted from the PINC AI Healthcare database, which includes 25% of US inpatient discharges.

**Results:**

Among 3494 patients (index TE: arterial, 69.1%; venous, 27.1%; both, 3.7%), mean (SD) age was 70.7 (14) years, and most patients were male (58.6%), White (81.2%), with Medicare insurance (72.6%). Mean (SD) Charlson Comorbidity Index score was 3.2 (2.3). Mean total hospitalization costs were $24 403 during the index hospitalization (mean [SD] hospital length of stay [LOS], 7 [9] days). A third (*n* = 1150) of patients were admitted to the intensive care unit (mean cost, $29 342; mean [SD] LOS, 5 [7] days). During 30 days and 2 years of follow-up, the TE-related readmission rate was 6.4% and 20.0%, respectively. All-cause mortality was 6.2% during index hospitalization; an additional 4.7% occurred during the 2-year follow-up period.

**Conclusion:**

Among patients with PV and TE, inpatient hospitalization HCRU, costs, and mortality were substantial. These findings highlight the importance of preventing TEs in the management of PV.

## Graphical Abstract

**Graphical Abstract:**
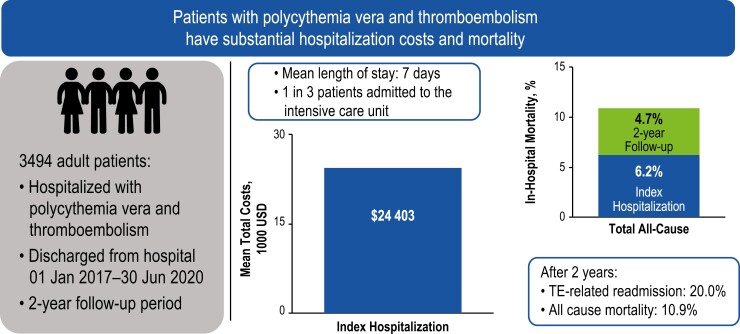

**Implications for Practice**

The findings from this retrospective analysis using records from a large US discharge database illustrate that inpatient care for patients with polycythemia vera (PV) who experience venous or arterial thromboembolic events (TEs) is associated with substantial resource use, costs, and mortality. The study findings highlight the importance of preventing TEs in the management of PV to reduce hospitalizations, morbidity, and mortality.

## Introduction

Polycythemia vera (PV) is a myeloproliferative neoplasm characterized by elevated hematocrit (Hct), trilineage myeloproliferation in the bone marrow, and presence of *Janus kinase (JAK)2* activating mutations.^1^ The estimated prevalence of PV in the United States is between 44 and 57 cases per 100 000 individuals, corresponding to ~150 000 to 200 000 US patients.^2,3^

Patients with PV have an increased risk of developing thromboembolic events (TEs) compared with the general population, including TEs in the venous and arterial vasculatures (VTE/ATE).^4-6^ With ~1 in 3 patient deaths related to TEs among patients with PV,^7^ they are the main contributor to a shortened life expectancy compared with the general population.^8-10^ As a result, lowering the risk of TEs is a primary treatment goal.^4-6,11,12^ The CYTO-PV study indicated that within a median of 31 months of follow-up, patients with high Hct (≥45%) had elevated rates of developing cardiovascular events and TEs compared with those with low Hct (<45%; hazard ratio: 2.69; 95% CI, 1.19-6.12).^12^ In real-world settings, where Hct control is less stringent, TE rates as high as 28% have been reported over similar time frames.^10^ In addition to TE and cardiovascular complications, PV is also associated with burdensome symptoms, including discomfort related to splenomegaly, fatigue, concentration problems, pruritus, night sweats, and bone pain.^13^ Management of PV to maintain Hct <45% and improve symptoms is primarily achieved through therapeutic phlebotomies, aspirin, and/or cytoreductive medical treatments.^14^

Prior data indicate that healthcare resource utilization (HCRU) and costs are often elevated in patients with PV due to its chronic nature and associated complications, with the co-occurrence of TEs leading to further increases in this patient population.^15,16^ Increased HCRU in both inpatient and outpatient settings contributes to the increased all-cause HCRU costs, with inpatient costs among patients with PV and TEs 4 times those among patients without TEs.^16^ However, the PV treatment landscape has evolved over the last decade with the addition of the JAK1/JAK2 inhibitor ruxolitinib and new interferon formulations,^14^ and contemporary data are needed regarding the impact of TEs on inpatient HCRU and costs in PV. This study aimed to describe HCRU, costs, and mortality that occurred during TE-related hospitalizations among patients with PV in a contemporary real-world setting in the United States.

## Patients and methods

### Study design and patients

**Figure 1.**
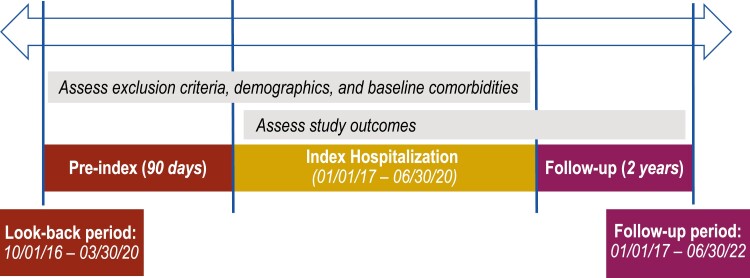
Study timeline.

This was a retrospective cohort study based on data abstracted from the PINC AI Healthcare database, a large, geographically diverse hospital discharge database that includes 25% of US inpatient discharges. Hospitals in the database represent the 4 geographic US regions with distribution as follows: Northeast, 12.9%; Midwest, 25.5%; South, 41.8%; and West, 19.8%. The study included adult inpatients (age ≥ 18 years) with PV and with predefined TEs (VTEs: deep vein thrombosis, pulmonary embolism, or superficial thromboembolism; ischemic stroke, myocardial infarction, transient ischemic attack, or peripheral arterial thrombosis) who were discharged from US hospitals between January 1, 2017, and June 30, 2020. Patients with PV and TE diagnoses were identified by International Classification of Diseases, Tenth Revision (ICD-10) coding (Supplementary Table S1). Discharge diagnosis could be principal or secondary for both PV and TE. If a patient had multiple visits meeting the selection criteria, the earliest visit was considered the index visit (Figure 1). The analysis included a 2-year follow-up period after first hospitalization for TE during the study period (index visit) and a 90-day preindex period. Patients from hospitals without continuous data submission for the preindex, index, and follow-up periods were excluded.

### Ethics statement

Given the use of deidentified data exclusively (Health Insurance Portability and Accountability Act compliant), the study was deemed not to require institutional ethics board review based on the policy of the Office of Human Subjects Research Protections, National Institutes of Health, under the revised Common Rule.

### Assessments

Patient demographics and clinical characteristics were assessed at the index visit. Comorbidities were assessed during the index visit and 90-day preindex period. HCRU and costs of TE-related hospitalizations, hospital length of stay (LOS), intensive care unit (ICU) admission, ICU LOS, hospital readmission risk (during the 30 days, 90 days, 1 year, and 2 years after the index visit), and in-hospital mortality were assessed at the index visit and for any readmissions during the 2-year follow-up period. Total costs of index hospitalization and costs of ICU care were not mutually exclusive (ie, total index costs comprised all costs incurred during index hospitalization, regardless of whether they involved an ICU stay, whereas total ICU costs comprised any costs related to an ICU admission, including ICU costs incurred during the index hospitalization).

### Statistical analyses

Costs were adjusted to 2021 US dollars based on the medical care component of the Consumer Price Index for Inpatient Services and were reported directly by the hospitals with comprehensive accounting systems based on relative value units or ratio of cost to charges (RCC) methods or converted from charges submitted by hospitals to cost based on RCC by Premier Inc. All costs were validated and reconciled at the department level against each hospital’s financial report by Premier Inc. Between-group comparisons using unadjusted univariate statistics were performed for patients with ATEs, VTEs, or both at index and during follow-up. Between-group comparisons for categorical variables were evaluated using chi-square or Fisher tests, and 2-sample comparisons for continuous variables were evaluated using a *t*-test or Wilcoxon rank sum test.

## Results

### Patient characteristics

**Figure 2.**
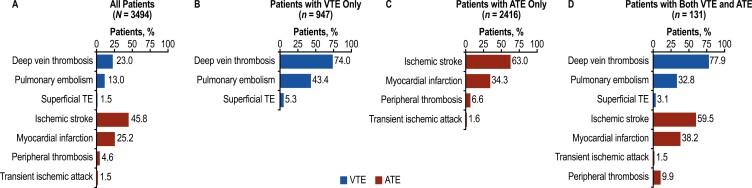
Thromboembolic events at index hospitalization. Percentage of patients with different types of TE among (A) all patients, (B) patients with VTE only, (C) patients with ATE only, and (D) patients with both VTE and ATE. TEs were preselected and defined in the protocol a priori. ATE, arterial thromboembolic event; TE, thromboembolic event; VTE, venous thromboembolic event. Percentages add to >100% because patients could have >1 TE.

A total of 3494 patients with PV and a TE were included in the analysis after applying inclusion criteria (Supplementary Figure S1). Mean age was 70.7 years, and most patients were male (58.6%), White (81.2%), and had Medicare as their primary insurance (72.6%; Table 1). All patients lived in the United States, almost half (45.4%) in the southern states, 20.6% in the Midwest, 18.6% in the Northeast, and the remaining 15.4% in the western states. Mean (SD) Charlson Comorbidity Index score was 3.2 (2.3). Overall, 69.1% (2416/3494) of patients had only ATEs at index hospitalization, 27.1% (947/3494) had only VTEs, and the remaining 3.7% (131/3494) had both. The most common TEs leading to hospitalization in the total population were ischemic stroke (45.8%), myocardial infarction (25.2%), deep vein thrombosis (23.0%), and pulmonary embolism (13.0%; Figure 2).

**Table 1.** Demographics, patient characteristics, and hospital characteristics at index in patients.

Parameter	Venous TE only (*n* = 947)	Arterial TE only (*n* = 2416)	Both (*n* = 131)	Total (*N* = 3494)
Mean (SD) age, years	70.0 (14.0)	71.0 (13.0)	71.7 (13.0)	70.7 (14.0)
Sex, *n* (%)				
Male	515 (54.4)	1458 (60.3)	75 (57.3)	2048 (58.6)
Female	432 (45.6)	957 (39.6)	56 (42.7)	1445 (41.4)
Unknown	0	1	0	1
Race, *n* (%)				
White	795 (83.9)	1943 (80.4)	99 (75.6)	2837 (81.2)
Black	69 (7.3)	207 (8.6)	11 (8.4)	287 (8.2)
Other	66 (7.0)	221 (9.1)	17 (13.0)	304 (8.7)
Unknown	17 (1.8)	45 (1.9)	4 (3.1)	66 (1.9)
Ethnicity, *n* (%)				
Hispanic	37 (3.9)	106 (4.4)	9 (6.9)	152 (4.4)
Non-Hispanic	706 (74.6)	1833 (75.9)	98 (74.8)	2637 (75.5)
Unknown	204 (21.5)	477 (19.7)	24 (18.3)	705 (20.2)
Health insurance type, *n* (%)				
Medicare	683 (72.1)	1756 (72.7)	99 (75.6)	2538 (72.6)
Medicaid	67 (7.1)	189 (7.8)	11 (8.4)	267 (7.6)
Private insurance	162 (17.1)	366 (15.1)	12 (9.2)	540 (15.5)
Uninsured	19 (2.0)	66 (2.7)	4 (3.1)	89 (2.5)
Other/unknown	16 (1.7)	39 (1.6)	5 (3.8)	60 (1.7)
Comorbidities^a^				
Myocardial infarction	85 (9.0)	981 (40.6)	58 (44.3)	1124 (32.2)
Congestive heart failure	236 (24.9)	693 (28.7)	43 (32.8)	972 (27.8)
Peripheral vascular disease	89 (9.4)	269 (11.1)	24 (18.3)	382 (10.9)
Cerebrovascular disease	18 (1.9)	1614 (66.8)	76 (58.0)	1708 (48.9)
Dementia	74 (7.8)	269 (11.1)	19 (14.5)	362 (10.4)
Chronic pulmonary disease	245 (25.9)	644 (26.7)	33 (25.2)	922 (26.4)
Diabetes, chronic	113 (11.9)	365 (15.1)	11 (8.4)	489 (14.0)
Diabetes, nonchronic	104 (11.0)	338 (14.0)	20 (15.3)	462 (13.2)
Renal disease	244 (25.8)	632 (26.2)	37 (28.2)	913 (26.1)
Any malignancy	153 (16.2)	237 (9.8)	20 (15.3)	410 (11.7)
Mean (SD) Charlson Comorbidity Index score	2.4 (2.5)	3.4 (2.1)	3.8 (2.6)	3.2 (2.3)
Hospital setting, *n* (%)				
Urban	832 (87.9)	2107 (87.2)	117 (89.3)	3056 (87.5)
Rural	115 (12.1)	309 (12.8)	14 (10.7)	438 (12.5)
Teaching status, *n* (%)				
Teaching	502 (53.0)	1309 (54.2)	80 (61.1)	1891 (54.1)
Nonteaching	445 (47.0)	1107 (45.8)	51 (38.9)	1603 (45.9)
Geographic region, *n* (%)				
South	444 (46.9)	1078 (44.6)	63 (48.1)	1585 (45.4)
Midwest	185 (19.5)	515 (21.3)	21 (16.0)	721 (20.6)
Northeast	183 (19.3)	440 (18.2)	28 (21.4)	651 (18.6)
West	135 (14.3)	383 (15.9)	19 (14.5)	537 (15.4)

TE, thromboembolic event.

^a^Any comorbidities incurring in ≥10% of the study population.

### HCRU and costs at index admission

**Figure 3.**
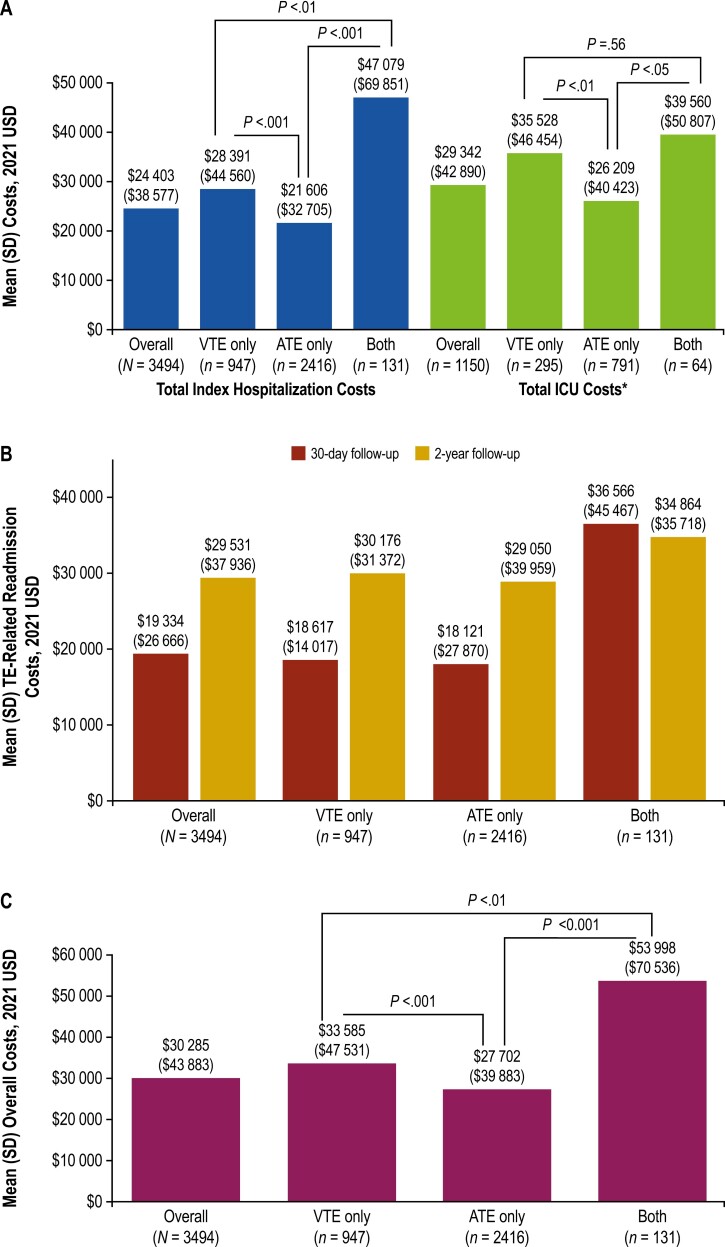
Costs of hospitalization. Mean (SD) costs of (A) total hospitalization and ICU at index visit, (B) TE-related hospital readmission during follow-up, and (C) combined total during index visit and TE-related readmissions during 2-year follow-up. ATE, arterial thromboembolic event; ICU, intensive care unit; TE, thromboembolic event; USD, US dollars; VTE, venous thromboembolic event. *Total index hospitalization costs and ICU costs are not mutually exclusive (ie, total index costs comprised all costs incurred during index hospitalization, regardless of whether they involved an ICU stay, whereas total ICU costs comprised any costs related to an ICU admission, including ICU costs incurred during the index hospitalization).

During index TE hospitalization, mean (SD) total hospitalization costs for the study population were $24 403 ($38 577, Figure 3A), and the mean (SD) hospital LOS was 7 (9) days. Total hospitalization costs were significantly different between patients with different types of vascular events at index (*P* <.01). Highest costs (mean [SD]) were reported for patients with both VTEs and ATEs at index ($47 079 [$69 851]), ~1.7 times higher than those with VTE alone ($28 391 [$44 560]) and 2.2 times higher than those with ATE alone ($21 606 [$32 705]). In the VTE-only group, total hospitalization costs were ~1.3 times higher than those for ATE only. A similar pattern was observed for the third of patients (*n* = 1150) in the study admitted to the ICU, with a mean (SD) ICU cost of $29 342 ($42 890; Figure 3A) and mean (SD) ICU LOS of 5 (7) days. Patients with both VTEs and ATEs had the highest (mean [SD]) costs for ICU visits ($39 560 [$50 807]), 1.1 times higher than those for patients with VTE only ($35 528 [$46 454]), which were 1.4 times higher than those for patients with ATE only ($26 209 [$40 423]). Patients with VTEs generally had longer ICU visits than those with ATEs: mean (SD) ICU LOS was 6 (8) days among patients with VTEs only (*n* = 295), 4 (6) days among patients with ATEs only (*n* = 791; *P* =.0001 vs VTEs only), and 6 (5) days among patients with both VTEs and ATEs (*n* = 64; *P* =.79 vs VTEs only).

### HCRU and costs of TE-related readmissions

The TE-related readmission rate in the study population was 6.4% during 30 days of follow-up and 20.0% during 2 years of follow-up, with a similar pattern observed in the 3 TE subgroups (Table 2). Mean (SD) TE-related hospital readmission costs increased ~1.5-fold from $19 334 ($26 666) at 30 days of follow-up to $29 531 ($37 936) at 2 years (Figure 3B). This pattern was observed in the overall study population and among patients with VTEs or ATEs only. For patients who had both VTEs and ATEs, the cost of readmission at 30 days was ~2 times higher than that for patients with VTE or ATE only and did not increase further at 2 years.

**Table 2.** TE-related hospital readmission rate during follow-up.

	Cumulative readmission rate, *n* (%)			
Follow-up time	Venous TE only (*n* = 947)	Arterial TE only (*n* = 2416)	Both (*n* = 131)	Total (*N* = 3494)
30 days	57 (6.0)	152 (6.3)	13 (9.9)	222 (6.4)
90 days	86 (9.1)	246 (10.2)	17 (13.0)	349 (10.0)
1 year	125 (13.2)	402 (16.6)	21 (16.0)	548 (15.7)
2 years	163 (17.2)	509 (21.1)	26 (19.8)	698 (20.0)

TE, thromboembolic event.

Combining costs accrued at index hospitalization and at TE-related readmissions over the 2-year follow-up period, total mean (SD) inpatient hospital cost in the study population was $30 285 ($43 883; Figure 3C). Patients with both VTEs and ATEs at index had a significantly (*P* <.01) greater total mean inpatient hospital cost ($53 998 [$70 536]), 1.6 times higher than those for patients with VTEs only ($33 585 [$47 531]), which was 1.2 times higher than those for patients with ATEs only ($27 702 [$39 883]).

### In-hospital mortality

**Figure 4.**
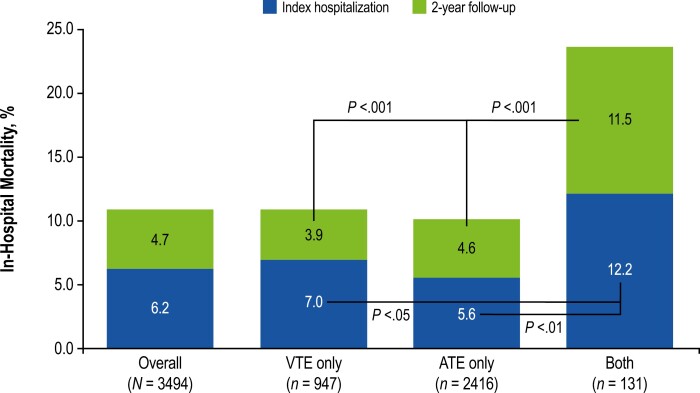
Total all-cause in-hospital mortality rate at index visit and 2-year follow-up. ATE, arterial thromboembolic event; VTE, venous thromboembolic event.

All-cause in-hospital mortality during index hospitalization was 6.2% in the total population, with an additional 4.7% occurring during the 2 years following index hospitalization (Figure 4). The mortality rate at index was significantly higher among patients with both VTEs and ATEs at index (12.2%) versus those with VTEs (7.0%; *P* <.05) or ATEs alone (5.6%; *P* <.01). During the 2 years following index hospitalization, the mortality rate remained significantly higher among patients with both ATEs and VTEs at index (11.5%) versus those with VTEs only (3.9%; *P* <.001) and ATEs only (4.6%; *P* <.001).

## Discussion

This retrospective cohort study demonstrated that TEs are associated with high levels of inpatient HCRU, costs, and mortality in patients with PV. In particular, the ICU admission rate, occurring in approximately one-third of patients, is indicative of the severity of TEs and their effects in patients with PV. The impact on HCRU, costs, and mortality was greatest for a subset of patients who presented with both VTEs and ATEs at index hospitalization, followed by those with VTEs only. The costs identified here are consistent with those reported previously in PV populations. An administrative claims analysis of US patients with PV who initiated hydroxyurea between 2005 and 2012 reported inpatient costs of ~$20 000 (2013 USD) in patients with a TE, ~4 times greater than inpatient costs for patients with PV without TEs in the same population.^16^ Similarly, a Japanese administrative claims analysis covering 2008-2015 reported that inpatient costs were more than doubled for patients with PV who had a TE compared with those without a TE (¥612 000 vs ¥245 000; ~$5087 vs $2036 when converted to 2015 USD).^15^ The costs reported here are higher than those identified in other non-PV populations with TEs in the United States. A previous analysis of the PINC AI Healthcare database that focused on hospitalized medically ill (nonsurgical) patients receiving thromboprophylaxis reported mean costs of index hospitalization that ranged between $15 814 and $20 282,^17^ which are lower than the costs required to manage patients with PV and TEs reported here. Similarly, US hospitalization costs of VTEs in patients without PV have been estimated to be between $1500 and $3000 per day^18^ and between $9000 and $15 000 overall,^19^ whereas ATEs cost between $15 000 and $20 000 overall.^20,21^

The readmission rate was 20% throughout the 2-year follow-up period, highlighting the long-term repercussions of thromboembolic complications in patients with PV, including an elevated risk for subsequent TEs among patients with PV with a history of thrombosis.^22^ These findings are again consistent with an ongoing need for hospital readmission described in other non-PV populations with TEs. In a retrospective claims analysis, 6-month VTE-related readmission rates were 2.1% for patients with a VTE at index hospitalization, 1.9% for those with heart failure, and 1.4% for those with ischemic stroke.^23^ A separate analysis of the Nationwide Readmissions database indicated that 17.5% of patients discharged following an index hospitalization with acute VTE required readmission within 30 days.^24^

The observed mortality rates during index hospitalization and the follow-up period reported here highlight the poor prognosis associated with TEs in patients with PV. TEs are a known prognostic factor for survival in patients with PV and are included as a key component of risk stratification.^25^ The mortality rates during initial hospitalization reported here often exceeded those reported by separate studies of non-PV populations hospitalized with TEs (VTE, 2%-6%^24,26^; acute ischemic stroke, 5%-6%^20^; peripheral arterial disease, 3%^21^), suggesting patients with PV who experience a TE may require additional or more active management compared with non-PV populations.

The treatment landscape of PV has shifted in recent years toward increased focus on disease modification and related clinical outcomes, including thrombosis-free survival.^27^ This is particularly relevant for adolescents and young adults with PV, who can experience devastating TEs early in the disease course, even at the time of PV diagnosis.^28^ The occurrence of TEs can lead to increased morbidity, time away from work, loss of income, and further health complications later in life, adding to the burden of disease for patients with PV.^10,15,29^ As part of the drive to improve disease modification and clinical outcomes for patients with PV, novel agents continue to be developed to treat patients with PV.^30^

Although VTEs and ATEs were observed in patients with PV in this study, ATEs alone were far more common than VTEs alone, occurring in over two-thirds of the population. Although this may reflect a methodologic limitation of this study (further discussed below), this aligns with reports from other recent real-world studies of PV populations.^10,31,32^ A retrospective analysis of a very large population (*N* = 50 405) of Medicare patients with PV between 2010 and 2017 reported comparatively high rates of ATEs (ischemic stroke, 46%; acute myocardial infarction, 30%; transient ischemic attack, 31%) versus VTEs (pulmonary embolism, 17%; deep vein thrombosis, 10%).^10^

Study limitations are typical of those related to the study design as an administrative database review. The database uses ICD-10 coding to identify patients with PV and TE, with the potential for inaccurate or incomplete coding. Because the PINC AI Healthcare database only captures hospital visits within the same system where the index hospitalization occurred, the readmission risk, mortality risk, and costs associated with the follow-up time periods might have been underestimated. Due to the focus of this analysis, more expansive medical details (eg, laboratory data or treatment information) were not included. Also, as the study was designed to assess inpatient costs only, the overall costs of TE management were not fully represented. Finally, the study focused on HCRU, costs, and mortality during TE-related hospitalizations among patients with PV, not incremental costs of PV, and therefore did not include a comparator group of non-PV patients with TE.

## Conclusions

This study provides important insights into the substantial healthcare burden associated with TE-related hospitalizations in patients with PV. The economic impact, risk of hospital readmissions, and elevated mortality rates underscore the importance of active patient management to improve the outcomes of patients with PV. Future research should focus on identifying optimal strategies for management of patients with PV to best prevent TEs in this high-risk population.

## Supplementary Material

oyaf001_suppl_Supplementary_Material

## Data Availability

The data underlying this article from the PINC AI Healthcare database (PHD) are proprietary and cannot be shared externally.
