# Supplementary material for: Resource utilization and inpatient hospitalization costs associated with thromboembolic events among patients with polycythemia vera
Source: Oncologist. 2025 Mar 4;30(2):oyaf001. doi: 10.1093/oncolo/oyaf001 (PMC11879438; doi:10.1093/oncolo/oyaf001)
Supplement: oyaf001_suppl_Supplementary_Material [file oyaf001_suppl_supplementary_material.docx]

**Supplementary Materials**

**Resource Utilization and Inpatient Hospitalization Costs Associated With Thromboembolic Events Among Patients With Polycythemia Vera**

Jingbo Yu, MD, PhD,^1^ Julie Gayle, MPH,^2^ Ning Rosenthal, MD, MPH, PhD,^2^ Harold Brown, MBA, MHA,^2^ Evan Braunstein, MD, PhD,^1^ Naveen Pemmaraju, MD^3^

^1^Incyte Corporation, Wilmington, DE, USA; ^2^PINC AI^™^ Applied Sciences, Premier Inc., Charlotte, NC, USA; ^3^Department of Leukemia, The University of Texas MD Anderson Cancer Center, Houston, TX, USA

**Contents**

Supplementary Table 1. Coding of Study Variables 2

Supplementary Figure 1. Patient Selection and Attrition 3

##### Supplementary Table 1. Coding of Study Variables

| **Comorbidity** | **Type** | **ICD-10 codes** |
| --- | --- | --- |
| Polycythemia vera | Dx | 45 |
| Cardiovascular and thromboembolic events |  |  |
| Myocardial infarction | Dx | I21.x, I22.x, I23.x, I25.2 |
| Congestive heart failure | Dx | I50.x |
| Peripheral vascular disease | Dx | I73.9, I71.00, I71.01, I71.02, I71.03, I71.1, I71.2, I71.3, I71.4, I71.5, I71.6, I71.8, I71.9, I96, Z95.828 |
|  | Proc | 04RK07Z, 04RK0JZ, 04RK0KZ, 04RK47Z, 04RK4JZ, 04RK4KZ, 04RL07Z, 04RL0JZ, 04RL0KZ, 04RL47Z, 04RL4JZ, 04RL4KZ, 04RM07Z, 04RM0JZ, 04RM0KZ, 04RM47Z, 04RM4JZ, 04RM4KZ, 04RN07Z, 04RN0JZ, 04RN0KZ, 04RN47Z, 04RN4JZ, 04RN4KZ, 04RP07Z, 04RP0JZ, 04RP0KZ, 04RP47Z, 04RP4JZ, 04RP4KZ, 04RQ07Z, 04RQ0JZ, 04RQ0KZ, 04RQ47Z, 04RQ4JZ, 04RQ4KZ, 04RR07Z, 04RR0JZ, 04RR0KZ, 04RR47Z, 04RR4JZ, 04RR4KZ, 04RS07Z, 04RS0JZ, 04RS0KZ, 04RS47Z, 04RS4JZ, 04RS4KZ, 04RT07Z, 04RT0JZ, 04RT0KZ, 04RT47Z, 04RT4JZ, 04RT4KZ, 04RU07Z, 04RU0JZ, 04RU0KZ, 04RU47Z, 04RU4JZ, 04RU4KZ, 04RV07Z, 04RV0JZ, 04RV0KZ, 04RV47Z, 04RV4JZ, 04RV4KZ, 04RW07Z, 04RW0JZ, 04RW0KZ, 04RW47Z, 04RW4JZ, 04RW4KZ, 04RY07Z, 04RY0JZ, 04RY0KZ, 04RY47Z, 04RY4JZ, 04RY4KZ |
| Cerebrovascular disease | Dx | I60.x, I61.x, I62.x, I63.x, I65.x, I66.x, I67.x, I68.x, I69.x, G45.x |

Dx, diagnosis; ICD-10, International Classification of Diseases, Tenth Revision; Proc, procedure.

##### Supplementary Figure 1. Patient Selection and Attrition

**
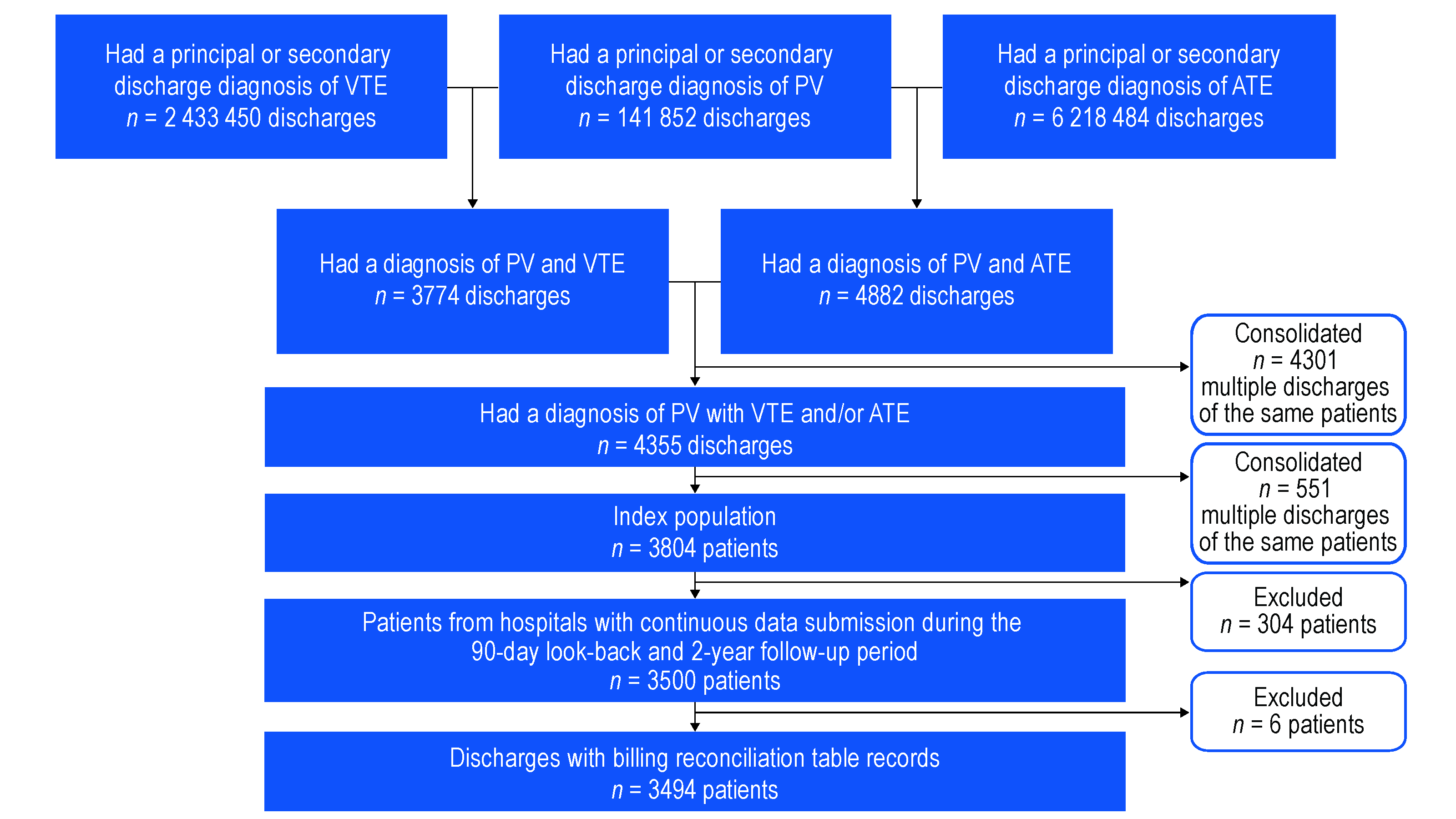
**

ATE, arterial thromboembolic event; PV, polycythemia vera; VTE, venous thromboembolic event.
